# The Association of Haptoglobin Gene Variants and Retinopathy in Type 2 Diabetic Patients: A Meta-Analysis

**DOI:** 10.1155/2017/2195059

**Published:** 2017-07-03

**Authors:** Huiqun Wu, Huan Wu, Lili Shi, Xinlu Yuan, Ying Yin, Mingjie Yuan, Yushan Zhou, Qianwen Hu, Kui Jiang, Jiancheng Dong

**Affiliations:** ^1^Department of Medical Informatics, Medical School of Nantong University, Nantong 226001, China; ^2^Department of Biomedical Engineering, University of Southern California, Los Angeles, CA 90089, USA; ^3^Department of Endocrinology, Affiliated Hospital of Nantong University, Nantong 226001, China

## Abstract

**Aims/Introduction:**

To collectively evaluate the association between haptoglobin (Hp) gene variants and diabetic retinopathy (DR) in patients with type 2 diabetes mellitus (T2DM).

**Methods:**

A comprehensive literature review was performed for eligible studies. After inclusion and exclusion selection as well as quality assessment, those studies meeting quality standards were included. In this study, diabetic patients with retinopathy were selected as the case group and those ones without DR were treated as the control group. The recessive model, allele model, additive model, heterozygote model, and homozygote model were utilized to investigate the association of three Hp gene variants and DR. Subgroup analysis on different severity of DR including nonproliferative diabetic retinopathy (NPDR) and proliferative diabetic retinopathy (PDR) was also conducted.

**Results:**

Six trials from different regions were finally included. A total of 1145 subjects containing 564 T2DM patients with retinopathy were included. The recessive model, allele model, additive model, and homozygote model results showed that Hp gene variants were not associated with DR, NPDR, and PDR. However, the heterozygote model indicated the association of Hp gene variants with DR.

**Conclusions:**

No association was found between the Hp gene variants and PDR and NPDR. More studies are required to verify these findings.

## 1. Introduction

Diabetes is a global health burden that affects populations' economic and health status. Due to the fact that diabetes is a system metabolic disorder, many organs and tissues will be affected and might be in dysfunction in the end. Management of diabetic complications has been a worldwide research of interest due to its clinical relevance and importance [1–3]. Vascular complications from diabetic complications such as nephropathy, retinopathy, and cardiovascular disease cause serious morbidity in patients with type 2 diabetes mellitus (T2DM) [4]. Diabetic retinopathy (DR) is one of the serious microvascular complications of T2DM patients and will finally cause blindness if not controlled effectively. As shown in recent epidemiologic studies, the prevalence of DR accompanied with T2DM is decreasing [5, 6].

Traditional measures to detect those microvascular complications mostly relied on imaging techniques. A digital fundus camera is such a device that could be utilized to record retinal blood vessels, the images of which are further assessed by ophthalmologists qualitatively. Besides biomarkers at the observable tissue level, some biomarkers at the molecular level are probably involved in the progression of above-mentioned microvascular complications. Heritability estimates for DR range from 25 to 50%, and the same degree of DR has been found in a cohort of identical twins with diabetes, suggesting that genetic variation is associated with DR. Haptoglobin (Hp) gene (rs137853233) is one of such candidate genes and is encoded by a single gene on chromosome 16. Hp gene has been discovered in human serum since 60 years ago, and the genetic mutation could be identified using SNP genotype data [7]. There are two common alleles for Hp which likely arose from a duplication event involving exons 3 and 4, usually denoted as Hp1 (containing 5 exons) and Hp2 (containing 7 exons) correspondingly. The most common type of allele variation consists of a major allele (M) and a minor allele (m); the Hp phenotype variants can be a major allele homozygote (Hp1-1), a heterozygote (Hp2-1), or a minor allele homozygote (Hp2-2). Hp could form stable complexes with plasma-free hemoglobin (Hb), as a result, blocking Hb-induced oxidative damage. The binding of Hp to apolipoprotein A1 (ApoA1) has also been reported to be related to the T2DM-associated cardiovascular disease [8]. Hp protein also facilitates the removal of Hb from the extravascular compartment via the CD163 macrophage scavenger receptor. Unlike Hp1-1 and Hp2-1, Hp2-2 exists as large circular polymers, having decreased binding affinity for free hemoglobin, and has been associated with biomarkers of oxidant stress andiron delocalization. Several longitudinal studies have recently demonstrated that Hp gene variants might be an independent risk factor for different kinds of diabetes complications such as diabetic retinopathy, cardiovascular disease, and nephropathy, but with the discrepancy in their findings [9–12]. Therefore, in this study, we conducted a meta-analysis to summarize the results by using a recessive model, allele model, additive model, heterozygote model, and homozygote model to investigate the association of Hp gene variants and DR.

## 2. Materials and Methods

### 2.1. Search Strategy

In the research, a comprehensive literature review was performed on four electronic databases: PubMed (National Center for Biotechnology Information), ISI (Web of Science), Embase, and CNKI (China National Knowledge Infrastructure), and the related studies published in English or Chinese before January 2017.While conducting the searches in the databases, no restrictions were imposed for time and language. Two subsets of citations were enhanced, namely, an indexing DR or diabetes, the other indexing Hp. For developing these subsets of citations, we used a combination of subject headings and text terms used in medical literature. Search terms used were as follows: (i) diabetic retinopathy, DR, diabetes without retinopathy, DWR, proliferative diabetic retinopathy, PDR, non-proliferative diabetic retinopathy, NPDR, metabolic syndrome, diabetic complication, diabetes, diabe^∗^, glycuresis; (ii) haptoglobin, Hp, Hp^∗^, gene, genetic, Hp1-1, Hp1-2, Hp2-1, Hp2-2; (iii) incidence, mortality, epidemiologic studies; and (iv) photography, photomicrography, photo$, image$, retinopathy, fundus. We combined the terms to generate a subset of citations that address the objective of our research study. We also hand searched reference lists of relevant articles for eligible studies. We examined the reference lists of all known primary and review articles to identify additional articles not captured by the electronic searches. The detailed search strategy is available from the authors. Two reviewers (Wu and Wu) independently examined the electronic searches and obtained full reports of all citations that were likely to meet the predefined selection criteria. Disagreements were resolved by consensus after discussion with a third reviewer (Yin).

### 2.2. Inclusion and Exclusion Criteria

The inclusion criteria were as follows: (i) case-control or cohort studies published about the association of Hp gene variants and DR in patients with the T2DM disease. (ii) Determination of DR was made by ophthalmoscope examination or fundus photography. (iii) Adequate information about the Hp genotype and allele were available. (iv) The language was written in English or Chinese.

The exclusion criteria were as follows: (i) insufficient data in frequencies of Hp genotype and allele, (ii) insufficient information about baseline characteristics of participants, (iii) literature reporting language other than English and Chinese, and (iv) patients diagnosed with T1DM other than T2DM.

### 2.3. Data Extraction and Analysis

In this study, those studies meeting the quality standards were included. The quality of the included studies was assessed by their risk of bias, directness, consistency of results, precision, publication bias, the magnitude of the effect, and so forth. In each of the included studies, three individual researchers (Wu, Wu, and Yuan) independently extracted the raw data associated with the values of Hp gene variants, DR numbers, total study numbers, ages, sex, and other factors. In instances where the raw data could not be extracted or calculated, we obtained the same by contacting the authors of these manuscripts. The recessive model, allele model, additive model, heterozygote model, and homozygote model were utilized to investigate the association of Hp gene variants and DR. For improving the accuracy of these tests, subgroup analyses were used to identify the test-related or other factors responsible for heterogeneity. In this study, RevMan (version: 5.2) was used to perform meta-analysis. Odds ratio (OR) and its 95% confidence interval (CI) were calculated for statistical analysis. Heterogeneity was established using chi-square and quantified by *I*^2^. In general, intergroup heterogeneity was evaluated by inconsistency index (*I*^2^) and heterogeneity. While the heterogeneity value was less than 0.1, pooled OR was estimated by using a random effect model or otherwise using a fix effect model. Sensitivity analyses were performed by omitting each study to identify possible study contributing to the heterogeneity. A two-sided value which is less than 0.05 means statistically significant. Funnel plots were utilized for investigating publication and other biases in meta-analysis.

## 3. Results

**Figure 1:**
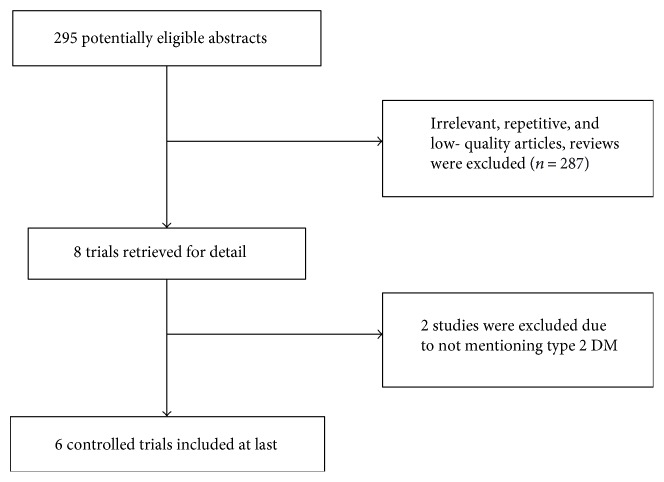
Flow chart of study selection.

The literature search yielded 295 references, six articles [13–18] out of which were eligible for inclusion finally.  Figure 1 outlines the study selection.

### 3.1. Summary Characteristics of Included Studies

A total of six trials were retrieved for detail data extraction (Table 1). The six studies from different regions including India, Finland, Ghana, China, and Brazil were finally included for this analysis. A total of 1145 subjects includes 564 type 2 DM patients accompanied with retinopathy. All studies met inclusion and exclusion criteria. The average age of the included population was comparable (the elders ranged from 51.27 to 69 years old). Hp allele frequencies of cases and controls are shown in Table 2. Meta-analysis was performed on all these studies after adjusting for different sorts of risk factors.

### 3.2. The Association of Hp Gene Variants and DR

**Figure 2:**
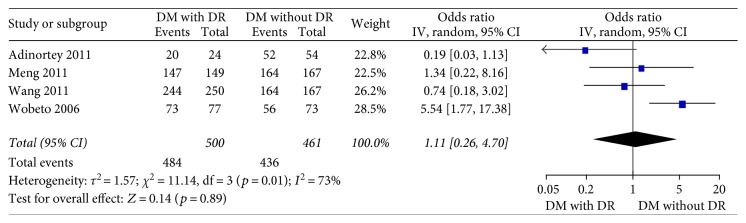
Forest plot for meta analysis comparing DR with DWR in dominant model (TT + CT versus CC).

**Figure 3:**
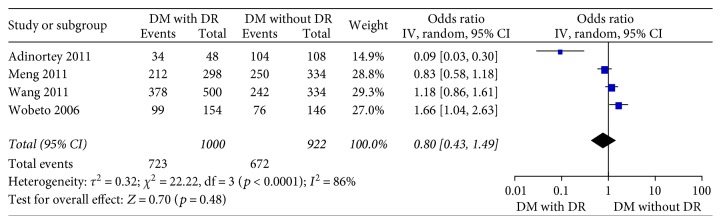
Forest plot for meta analysis comparing DR with DWR in allele model (T versus C).

**Figure 4:**
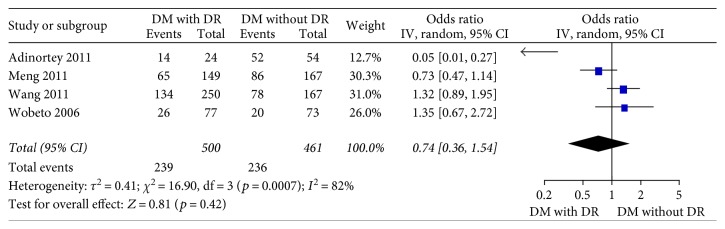
Forest plot for meta analysis comparing DR with DWR in recessive model (TT versus CT + CC).

**Figure 5:**
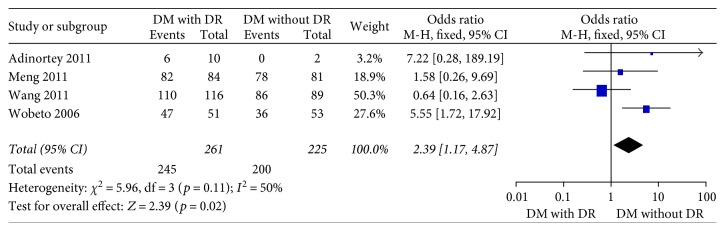
Forest plot for meta analysis comparing DR with DWR in heterozygote model (TC versus CC).

**Figure 6:**
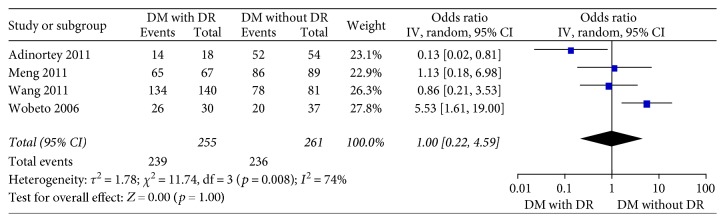
Forest plot for meta analysis comparing DR with DWR in homozygous model (TT versus CC).

Four studies [15–18] in the dominant model comparing those DWR and DR patients showed great heterogeneity of the studies (*X*^2^ *p* = 0.01 < 0.05, *I*^2^ = 73%); the total effect size OR in this study was 1.11 (95% CI: 0.26, 4.70), and the *Z* value was 0.14 (*p* > 0.05), suggesting that Hp gene variants were not associated with DR (Figure 2()). Similarly, the allelic model showed serious heterogeneity (*X*^2^ *p* < 0.05, *I*^2^ = 86%); the total effect size OR in this study was 0.80 (95% CI: 0.43, 1.49), and the *Z* value was 0.70 (*p* > 0.05), suggesting that no association was found between the Hp gene variants and DR (Figure 3()). The recessive model also showed serious heterogeneity of the studies (*X*^2^ *p* < 0.05, *I*^2^ = 82%); the total effect size OR and *Z* value were 0.74 (95% CI: 0.36, 1.54) and 0.81 (*p* > 0.05), respectively, indicating that Hp gene variant was not associated with DR either (Figure 4()). However, the heterozygote model showed no heterogeneity of the studies comparing DR and DWR (*X*^2^ *p* = 0.11 > 0.05, *I*^2^ = 50%); the total effect size OR in this study was 2.39 (95% CI: 1.17, 4.87), and the *Z* value was 2.39 (*p* < 0.05), suggesting that Hp gene variants were associated with DR (Figure 5()). The homozygous and additive model showed serious heterogeneity of the studies (*I*^2^ = 74% and 75%, resp.), and the *Z* value and the total effect size OR are both suggesting that Hp gene variants were not associated with DR (Figures 6() and 7()).

### 3.3. The Association of Hp Gene Variants and NPDR

**Figure 8:**
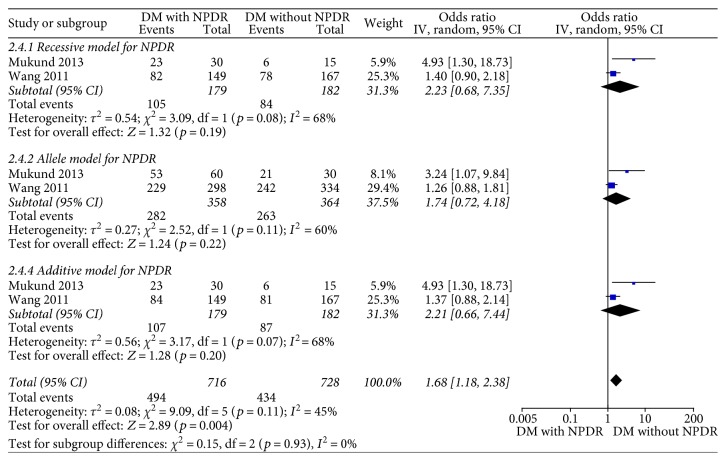
Forest plot for meta analysis comparing NPDR with DWR in different models.

In this study, two studies [13, 14] in the recessive model, allele model, and additive model all showed no heterogeneity of the studies between DR and DWR (*X*^2^*p* = 0.08, *I*^2^ = 68%; *X*^2^ *p* = 0.11, *I*^2^ = 60%; and *X*^2^ *p* = 0.07, *I*^2^ = 68%, resp.); the subtotal effect size OR in this study was 2.23 (95% CI: 0.68, 7.35), 1.74 (95% CI: 0.72, 4.18), and 2.21 (95% CI: 0.66, 7.44), respectively, all suggesting that Hp gene variants were not associated with NPDR (Figure 8). The heterozygote model and homozygous model were not suitable for the data; therefore, no results were obtained. However, the *p* value of the overall effect *Z* value from different models was less than 0.05, indicating the association between Hp gene variants and NPDR.

### 3.4. The Association of Hp Gene Variants and PDR

**Figure 9:**
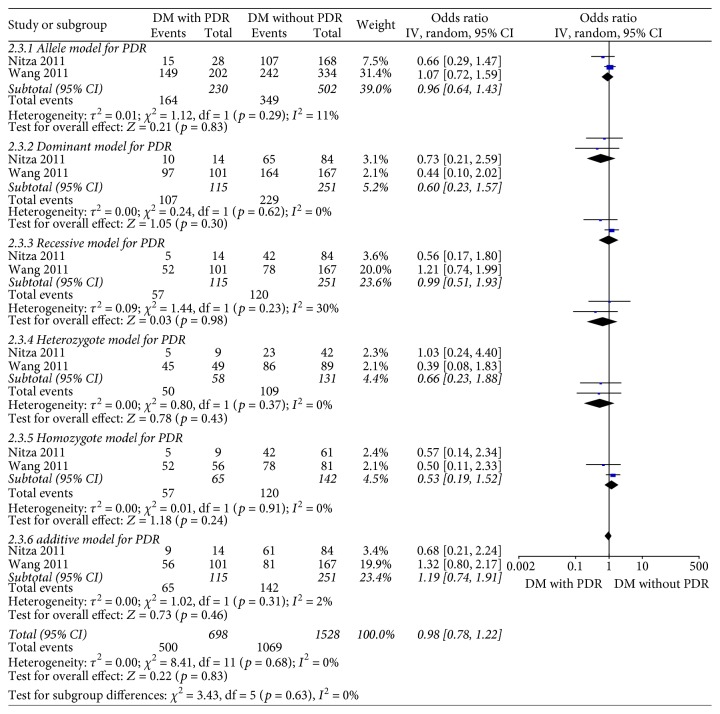
Forest plot for meta analysis comparing PDR with DWR in different models.

Two studies [14, 18] in all models showed no heterogeneity of the studies (*X*^2^ *p* = 0.29 > 0.1, *I*^2^ = 11%; *X*^2^ *p* = 0.62 > 0.1, *I*^2^ = 0%; *X*^2^ *p* = 0.23 > 0.1, *I*^2^ = 30%; *X*^2^ *p* = 0.37 > 0.1, *I*^2^ = 0%; *X*^2^ *p* = 0.91 > 0.1, *I*^2^ = 0%; and *X*^2^ *p* = 0.31 > 0.1, *I*^2^ = 2%), and the subtotal effect size OR in these studies was 0.96 (95% CI: 0.64, 1.43), 0.60 (95% CI: 0.23, 1.57), 0.99 (95% CI: 0.51, 1.93), 0.66 (95% CI: 0.23, 1.88), 0.53 (95% CI: 0.19, 1.52), and 1.19 (95% CI: 0.74, 1.91), respectively, all suggesting that Hp gene variants were not associated with PDR (Figure 9).

Funnel plots of each group of meta-analysis were shown, and those funnel plots without outlines were due to the use of the random effect model (See Supplementary Figures S1–S8 available online at https://doi.org/10.1155/2017/2195059).

## 4. Discussion

The global incidence and prevalence of DM have increased significantly over the last several decades [19, 20]. Patients with DM are often at a high risk of microvascular events [21, 22], in which DR is one of such risky events and might result in blindness at the late stage. The potential use of retinal vessel changes as a unique diagnostic biomarker for DR diagnosis is commonly investigated [23–25]. Recently, precision medicine idea empowers genetic codes as an early prediction tool for DR; therefore, we tried to analyze recent clinical studies on such possible gene candidates for DR prediction. Although several genome-wide association studies (GWAS) in different populations, such as Pima Indians [26, 27], Mexican-Americans [28], Asian [29–31], and Caucasians [32], identified multiple susceptibility loci to DR and reported evidence for the linkage of DR to several chromosomal regions, the susceptibility genes in these regions remain to be elucidated. Hp gene is one of such candidate genes and has been recently found to be related to vascular complications like retinopathy after diabetes since Hp-encoded protein is regarded as a positive acute phase reactant due to its binding capacity to hemoglobin (Hb) [33]. It has been figured out that free Hb is a relevant potent prooxidant which mediates several oxidative pathways resulting in the formation of hydroxyl radicals [34], which are often related to DM complications. Hp-Hb binding could exert an anti-inflammatory effect by removing heme compounds which catalyze the oxidation of arachidonic acid by prostaglandin synthase. Interestingly, such Hp-Hb binding capacity depends not only on serum Hp concentration but also on Hp phenotypes. Therefore, we investigated different Hp genotype variants and their association with DR complications. The recessive model, allele model, additive model, and homozygote model results showed that Hp gene variants were not associated with DR, NPDR, and PDR. However, the heterozygote model indicated the association of Hp gene variants with DR. The heterozygote model is the case where the heterozygote conveys both advantages and disadvantages, while both homozygotes convey a disadvantage. A well-established case of heterozygote advantage is that of the gene involved in sickle cell anaemia. A recent study [35] has shown that low levels of nitric oxide (NO), a major mediator of vascular tone, are significantly more prevalent in Hp2-2 DM individuals. The major reason for the reduced bioavailability of NO in the plasma Hp2-2 DM individuals is due to increased plasma Hb in these individuals. It has been reported that the Hp1-1 protein clears Hb more quickly than the Hp2-2 protein, and so there is more Hp2-2-Hb available to bind to ApoA1 in Hp2-2 individuals [36]. As Hp genotype was indicated to be associated with DR, there should be a tendency that such association should be consistent with the severity of the outcome in a dose-dependent manner, in our case, PDR. However, all models in our subgroup analysis showed no association between Hp gene variants and PDR. Nephropathy is usually regarded as one of the complications of T2DM at a serious stage and has been reported to be associated with PDR [37]. Nakhoul et al. reported the association between Hp gene and the risk of nephropathy [38]. However, such association was found to be not significant in Wobeto et al.'s study [39]; therefore, more studies are required to confirm the association between Hp genotype and severity of DR and the underlying mechanism is worth of investigation.

Meta-analysis is a comprehensive statistical method that has been used increasingly for combining and integrating data from a number of independent studies. However, the results of meta-analysis depend on the quality of primary researches included for further analysis. In this study, there are some limitations that might be affecting the summarized results. First, our studies included populations which are not from the same region and DM duration was not uniform in these research groups. Second, some publication bias could be attributed to the inaccuracy association between Hp gene and NPDR, therefore making its result not robust. Third, despite the relatively large size of the population from which cases were derived, the number of DR patients was relatively small. Furthermore, we had problems with missing data during data extraction in Hp1-1 phenotype subjects for NPDR, which have unpredictable effects on multivariate estimates on risk.

Despite the above-mentioned weaknesses, in the meta-analysis comparing DR with DWR, the heterozygote model indicated the association of Hp gene variants with DR. More primary large-sample and well-controlled studies on the association between Hp genotype variants and DR are required to verify these findings.

## Supplementary Material

Figure S1: Funnel plot for Meta analysis comparing DR with DWR in dominant model (TT+CT vs CC). Figure S2. Funnel plot for Meta analysis comparing DR with DWR in allele model (T vs C). Figure S3. Funnel plot for Meta analysis comparing DR with DWR in recessive model(TT vs CT + CC). Figure S4. Funnel plot for Meta analysis comparing DR with DWR in heterozygote model(TC vs CC). Figure S5. Funnel plot for Meta analysis comparing DR with DWR in homozygous model (TT vs CC). Figure S6. Funnel plot for Meta analysis comparing DR with DWR in additive model(TT + CC vs CT). Figure S7. Funnel plot for Meta analysis comparing NPDR with DWR in different models. Figure S8. Funnel plot for Meta analysis comparing PDR with DWR in different models.

## Figures and Tables

**Figure 7:**
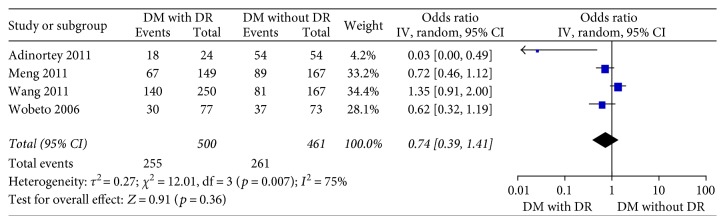
Forest plot for meta analysis comparing DR with DWR in additive model (TT + CC versus CT).

**Table 1:** The characteristics of included studies.

Study	Year	Country	Number of DR/DWR	Age (DWR)	Age (DR)	Male/female (DR)	Male/female (DWR)	DM duration (years) DR/DWR	BMI (kg/m^2^)	HbA1c (%)
Mukund et al.	2013	India	30/15 (NPDR)	55.93 ± 17.85	55.7 ± 11.97	16/14	7/8	11.6 ± 4.77/7.6 ± 3.59	—	—
Nitza et al.	2011	Finland	14/84 (PDR)	69 ± 9.7	62 ± 7.8	13/1	28/56	4.3 ± 3.7/19 ± 8	28.8 ± 2.5/28.7 ± 5.3	8.7 ± 2.5/7.4 ± 1.2
Adinortey et al.	2011	Ghana	24/73	52.70 ± 1.49 (male)	56.83 ± 0.97 (male)	101/189	46/62	—	26.86 ± 0.51 (male)	
				51.27 ± 1.48 (female)	53.36 ± 0.74 (female)				28.58 ± 0.40 (female)	
Wobeto et al.	2006	Brazil	97/73	—	—	—	—	18.0 ± 5.9/14.6 ± 4.5 (Hp1-1)	—	8.7 ± 1.9/8.4 ± 1.9 (Hp1-1)
								16.8 ± 6.6/13.8 ± 4.2 (Hp2-1)	—	8.8 ± 2.0/7.8 ± 1.9 (Hp2-1)
								18.8 ± 6.9/14.7 ± 3.7 (Hp2-2)	—	9.3 ± 1.9/8.3 ± 2.2 (Hp2-2)
Meng et al.	2011	China	149/168	58 ± 10	57 ± 11	82/67	91/77	12/9	26.3 ± 3.7/26.7 ± 4.0	8.77 ± 2.00/8.44 ± 1.91
Wang et al. (PDR)	2011	China	101/168	58.2 ± 10.6	52.8 ± 10.5	42/59	91/77	12.5/9	27.0 ± 3.9/26.7 ± 4.0	7.84 ± 2.13/8.44 ± 1.91
(NPDR)			149/168		57.0 ± 11.1	82/67	91/77	12.0/9	26.3 ± 3.7/26.7 ± 4.0	8.77 ± 2.00/8.44 ± 1.91

**Table 2:** Hp allele frequencies of cases and controls.

Study	DR			DWR		
	CC	CT	TT	CC	CT	TT
	HP1-1 (%)	HP2-1 (%)	HP2-2 (%)	HP1-1 (%)	HP2-1 (%)	HP2-2 (%)
Mukund et al.	0 (0)	7 (23.3)	23 (76.7)	0 (0)	9 (60.0)	6 (40.0)
Nitza et al.	4 (28.6)	5 (35.7)	5 (35.7)	19 (22.6)	23 (27.4)	42 (50.0)
Adinortey et al.	4 (16.7)	6 (25.0)	14 (58.3)	2 (3.7)	0 (0)	52 (96.3)
Wobeto et al.	4 (5.2)	47 (61.0)	26 (33.8)	17 (23.3)	36 (49.3)	20 (27.4)
Meng et al.	2 (1.4)	82 (55.0)	65 (43.6)	3 (1.8)	78 (46.7)	86 (51.5)
Wang et al. (PDR)	4 (4.0)	45 (44.6)	52 (51.4)	3 (1.8)	86 (51.5)	78 (46.7)
(NPDR)	2 (1.4)	65 (43.6)	82 (55.0)	3 (1.8)	86 (51.5)	78 (46.7)
