# Supplementary material for: The Association of Haptoglobin Gene Variants and Retinopathy in Type 2 Diabetic Patients: A Meta-Analysis
Source: J Diabetes Res. 2017 Jul 3;2017:2195059. doi: 10.1155/2017/2195059 (PMC5512055; doi:10.1155/2017/2195059)
Supplement: Supplementary file 1 — Figure S1: Funnel plot for Meta analysis comparing DR with DWR in dominant model (TT+CT vs CC). Figure S2. Funnel plot for Meta analysis comparing DR with DWR in allele model (T vs C). Figure S3. Funnel plot for Meta analysis comparing DR with DWR in recessive model(TT vs CT + CC). Figure S4. Funnel plot for Meta analysis comparing DR with DWR in heterozygote model(TC vs CC). Figure S5. Funnel plot for Meta analysis comparing DR with DWR in homozygous model (TT vs CC). Figure S6. Funnel plot for Meta analysis comparing DR with DWR in additive model(TT + CC vs CT). Figure S7. Funnel plot for Meta analysis comparing NPDR with DWR in different models. Figure S8. Funnel plot for Meta analysis comparing PDR with DWR in different models. [file 2195059.f1.docx]

**Figure S1**| Funnel plot for Meta analysis comparing DR with DWR in dominant model (TT+CT vs CC)

**Figure S2**| Funnel plot for Meta analysis comparing DR with DWR in allele model (T vs C)

**Figure S3**| Funnel plot for Meta analysis comparing DR with DWR in recessive model(TT vs CT + CC)

**Figure S4**| Funnel plot for Meta analysis comparing DR with DWR in heterozygote model(TC vs CC)

**Figure S5**| Funnel plot for Meta analysis comparing DR with DWR in homozygous model (TT vs CC)

**Figure S6**| Funnel plot for Meta analysis comparing DR with DWR in additive model(TT + CC vs CT)

**Figure S7**| Funnel plot for Meta analysis comparing NPDR with DWR in different models

**Figure S8**| Funnel plot for Meta analysis comparing PDR with DWR in different models

Figure S1 Figure S2 Figure S3 Figure S4

Figure S5 Figure S6 Figure S7 Figure S8
